# Music-of-light stethoscope: a demonstration of the photoacoustic effect

**DOI:** 10.1088/0031-9120/51/4/045015

**Published:** 2016-06-07

**Authors:** D I Nikitichev, W Xia, E Hill, C A Mosse, T Perkins, K Konyn, S Ourselin, A E Desjardins, T Vercauteren

**Affiliations:** 1 Translational Imaging group, Centre for Medical Image Computing, University College London, London, UK; 2 Department of Medical Physics and Biomedical Engineering, University College London, Gower Street, WC1E 6BT, London, UK; d.nikitichev@ucl.ac.uk

## Abstract

In this paper we present a system aimed at demonstrating the photoacoustic (PA) effect for educational purposes. PA imaging is a hybrid imaging modality that requires no contrast agent and has a great potential for spine and brain lesion characterisation, breast cancer and blood flow monitoring notably in the context of fetal surgery. It relies on combining light excitation with ultrasound reception. Our brief was to present and explain PA imaging in a public-friendly way suitable for a variety of ages and backgrounds.

We developed a simple, accessible demonstration unit using readily available materials. We used a modulated light emitting diode (LED) torch and an electronic stethoscope. The output of a music player was used for light modulation and the chest piece of the stethoscope covered by a black tape was used as an absorbing target and an enclosed chamber.

This demonstration unit was presented to the public at the Bloomsbury Festival *On Light* in October 2015. Our stall was visited by over 100 people of varying ages. Twenty families returned in-depth evaluation questionnaires, which show that our explanations of the photoacoustic effect were well understood. Their interest in biomedical engineering was increased.

## 1. Introduction

The photoacoustic (PA) effect was discovered in 1880 by Alexander Graham Bell. He observed that when a beam of sunlight is focussed on a dark sample and rapidly interrupted with a rotating disk, sound is produced. This observation led him to the invention of the ‘photophone’ for transmitting the human voice [1]. In the past the PA effect has been vastly explored in solids for spectroscopy [2] allowing measurement of properties of materials such as absorption [3] and the speed of sound [4].

**Figure 1.**
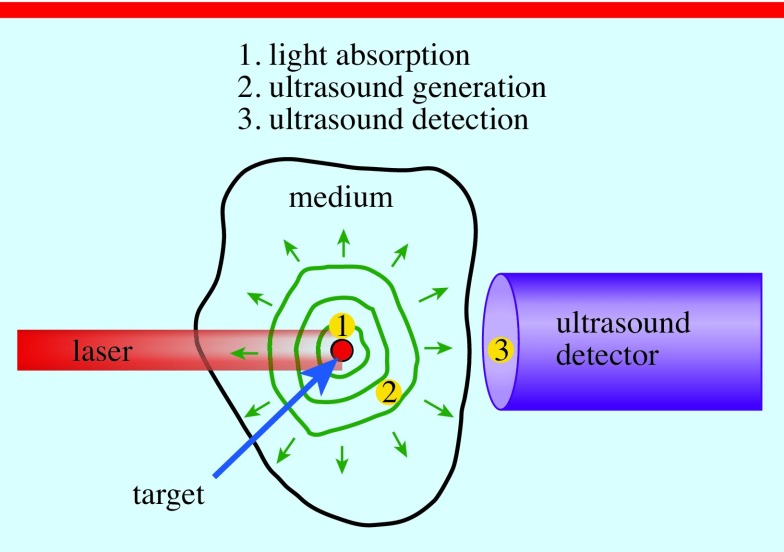
PA imaging can be explains as follows (1): the target (tissue) absorbed the modulated light (2), absorption leads to local temperature rise and as the results to the generation of ultrasound waves (3); high-quality ultrasound images are formed via ultrasound detector.

In recent years, the PA effect was investigated for medical imaging purposes, e.g. spine and brain lesion characterisation, lipid-rich plaques assessment, breast cancer diagnosis [5–11], and blood flow monitoring notably in the context of fetal surgery [12]. Thee traditional optical imaging techniques such as diffuse optical tomography and optical microscopy are limited in penetration depth or spatial resolution due to light scattering within the tissue. This limitation can be overcome by using PA imaging where ultrasound and optical techniques are combined. PA is a physical process, which relies on very brief pulses of light being sent into the tissue to provide detailed feedback on its structure. PA imaging is an emerging technique that includes three major steps: (1) Light absorption; (2) Ultrasound (acoustic) wave generation; (3) Ultrasound detection (figure 1). The main advantage of this approach is that PA signal is robust to scattering, reflection, and transmission conditions thereby improving the penetration depth and spatial resolution that can be used for vasculature imaging including placement on nerves, veins and arteries [13].

Firstly, the light that is modulated or generated from a pulsed laser is delivered to the biological target. As the light is absorbed in a sample it leads to a safe local increase of the temperature by ~1 K. This causes a very brief thermal expansion, which generates a pressure wave that propagates through the sample and can be detected as an acoustic wave.

Finally, ultrasound images of the organs, vessels or tissue are formed as a result of different light absorption spectra. These images contain additional information on the illuminated target. The University College London (UCL) GIFT-Surg project (Guided Instrumentation for Fetal Therapy and Surgery, www.gift-surg.ac.uk) was set up to research into devices using this technique to advance the field of fetal surgery and also provide public engagement for our work.

Recently, we demonstrated a proof-of-concept of the potential use of this technology to guide treatments of twin-to-twin transfusion syndrome, where a detailed view of the internal blood vessels and anatomy in the placenta is of critical importance [12, 14, 15]. This process is being built into the novel fetoscopic devices being developed under the project to advance the field of fetal surgery, where such detailed information is required.

Although this process involves light, we originally found PA imaging to be difficult to present and explain in a public friendly way suitable to a variety of age ranges and backgrounds. Inspired by the fact that phones have successfully been used in teaching physics to determine the gravitational acceleration [16] as well as to analyse the acoustic Doppler Effect [17], we decided to design a hands-on device illustrating the PA concept but decoupling it from imaging. We thought this would simplify the experimental setup and may enhance the public or students’ understanding.

Several PA demonstration kits have previously been developed using everyday items such as glass pickle jars, and wine bottles [18–20], which can absorb light and produce a humming sound. We wished to go further and use PA to change light into sound to produce recognisable music.

In this paper, we present a ‘Music-of-Light Stethoscope’ which uses the same scientific process but produces music rather than a simple humming noise. It converts audio frequency into light signals and back to audio frequencies, which the users can hear. The key components are only a stethoscope and a light torch. This demonstration kit was used both for teaching in the class room as well as in public engagement activities at UCL during The Universities and Colleges Admissions Service (UCAS) days [21] and the Bloomsbury Festival [22] to promote Science Technology Engineering & Math (STEM) subjects to the younger generations.

## 2. Experiment description

**Figure 2.**
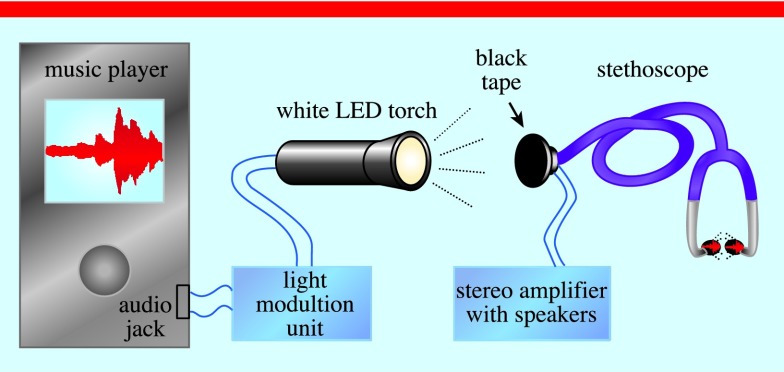
The experimental setup of a musical stethoscope that demonstrates a PA effect.

The complete demonstration kit consists of a music player, a light modulation unit (LMU), a white light emitting diode (LED, U2-1A Cree, 6500–7000 K, 700 Lm, fasttech, USA), a (optionally electronic) stethoscope (ES-120 Doctors Orders, Japan) and an optional stereo amplifier with speakers. A schematic of the system is given in figure 2.

In order to modulate the LED, a standard audio signal from a music player (PC, smartphone, etc) is sent via audio jack to a LMU where it is amplified and voltage biased. The LMU drives a white LED-based high power torch. This LED torch is used to illuminate black tape (0.2 mm thick), which generates a PA signal. The tape covers the chest piece of the stethoscope which is used to detect the PA signal. It is possible to listen to the signal directly with the stethoscope, or, if an electronic stethoscope is used, the system can be connected to a stereo amplifier with speakers so that a group of people can hear the reproduced sound.

**Figure 3.**
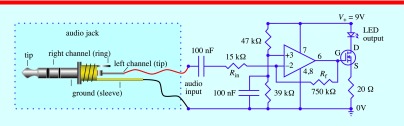
LMU includes audio signal amplification and voltage bias stages.

The LMU consisted of a voltage divider; a high-pass and a low-pass filters and an inverting amplifier (figure 3). The voltage divider was built using a pair of resistors (47 kΩ and 39 kΩ), and the high and low-pass filters were built each using a 100 nF capacitor. An inverting amplifier with a Gain of 50 (equation (1)) was constructed using an operational amplifier (OP-AMP TLC271IP, 1.7 MHz, DIP-8, TEXAS Instruments, USA) and two resistors (750 kΩ and 15 kΩ):

1 \begin{eqnarray*}\text{Gain}(Av)=\frac{{{V}_{\text{out}}}}{{{V}_{\text{in}}}}=\frac{-{{R}_{\text{f}}}}{{{R}_{\text{in}}}}\end{eqnarray*}

where *V*_out_ is the output voltage, *V*_in_ is the input voltage, *R*_f_ is the feedback resistor and *R*_in_ is the input resistor. In addition, a MOSFET transistor (IRLD014PBF, 1.7 A, 60 V, N Channel, Farnell, UK) with a 20 Ω 10 W resistor was added to drive the high current LED. The total cost of the system was £400 but it can be significantly reduced (~£100) if a regular stethoscope is used without stereo amplifier: £250 electronic stethoscope, £40 LMU, £15 LED torch, £20 cables and connectors, £30 stereo amplifier, £45 for a power supply and speakers.

## 3. Music-of-light stethoscope

The developed device is compatible with any standard headphone jack and allows members of the audience to play their favourite songs through the device, encouraging personal involvement and interest in the scientific content.

**Figure 4.**
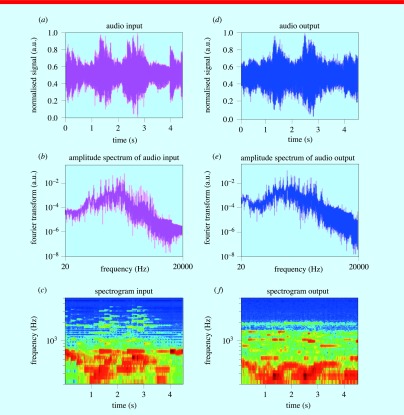
The comparison of original audio signal produced by a player with the output amplified PA signal: (a), (d) time and (b), (e) frequency domain input and output signals respectively; (c) spectrogram of input and (f) output of the part of the song ‘Für Elise’ by Beethoven.

As the torch is brought closer to the target (the black tape), the music produced by the system becomes louder. When the light is OFF, there is no music. In figure 4, audio input and output signals in time and frequency domains from the demonstration setup are shown.

The waveform of the original audio input signal produced by a player is compared with the amplified PA signal played through the speakers (4.5 s of a song ‘Für Elise’ by Beethoven). Both waveforms were recorded using WavePad Sound Editor program and displayed using Matlab. As it can be seen the PA signal looks similar to the original signal and also sounds similar (listen to the recorded PA signal in the provided supplement file stacks.iop.org/PhysEd/51/045015/mmedia). The spectrograms were generated by custom script in Matlab and compared in figures 4(c) and (f). As expected, the output spectrogram was noisier than the input, due to optical and audio noise.

## 4. Technical background

PA signal can be generated within an enclosed chamber when chopped light impinges on a solid in a chamber as explained by the Rosencwaig–Gersho theory [23].

The main criteria for PA signal generation are thermal and stress confinements. The thermal confinement refers to the heat generated by absorption of a short laser pulse (*τ*_p_), or its equivalent modulated light, and dissipated to the surrounding areas by thermal conduction (*τ*_th_) (equation (1)) [24, 25]. The thermal confinement condition is expressed by:

2 \begin{eqnarray*}{{\tau}_{\text{p}}}&lt;{{\tau}_{\text{th}}}\sim {{d}^{2}}/{{D}_{\text{T}}}\end{eqnarray*}

where *d* is the light penetration and *D*_T_ is the thermal diffusivity.

The stress confinement condition can be express as follows:

3 \begin{eqnarray*}{{\tau}_{\text{p}}}&lt;{}^{d}/{}_{{{v}_{\text{s}}}}\end{eqnarray*}

where *v*_s_ is the speed of sound in the material.

In PA imaging, high-energy laser pulses of around a nanosecond are typically used. Thus both thermal and stress confinement conditions are met. A short laser pulse (nanosecond) can deposit thermal energy in the target faster than thermal diffusion can dissipate that energy. Equally a very short laser pulse can induce a stress in the target fast enough to avoid stress-wave propagation. As a result, the PA signal is maximized.

In our experiment, when the light was modulated at less than 1 KHz (equivalent ~1 ms pulse) the thermal and stress confinement conditions were not met. The acoustic wave generation mechanism was thus different from standard PA signal generation used for PA imaging. Even so, as can still be explained by the Rosencwaig–Gersho theory [23], it worked well enough for our demonstration. As the modulated light shone on the black tape it was heated and cooled periodically. A thin layer of the nearby air (~1–2 mm for 1 KHz modulation) enclosed in the chest piece of the stethoscope responded thermally to the periodic heat flow [3, 23]. Thus the main source of acoustic signal was the cyclic heat flow from the tape to the enclosed nearby air. The incident light modulation frequency determined the frequency contents of the resulting acoustic signals, and the amplitudes of the signals were proportional to the amplitude of the light.

## 5. Evaluation of our system during a public engagement activity

The GIFT-Surg project is for developing advanced surgical tools and novel imaging techniques for breakthrough transformations and improvements in the treatment of congenital problems in the womb. It has a strong commitment to public engagement in order to raise awareness of the fields of fetal surgery, image-guided interventional surgery and engineering in healthcare to current school-age children and the wider public. Object-based learning has been demonstrated to enhance young people’s interest in and understanding of subjects being taught [26]. Objects provide a direct link to the topic, learners use both sight and touch senses, which helps drawing conclusions. The PA demonstration kit we developed provides a stimulating way of teaching pupils (e.g. secondary school students) some basic concepts of physics, including the transformation of electricity to light, to heat, to mechanical expansion and so to sound, and also provides a motivation to select studies across STEM subjects.

**Figure 5.**
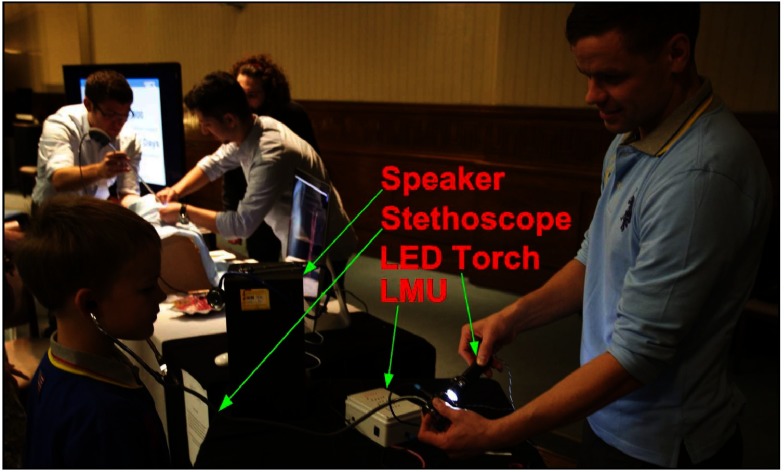
A photograph of an 8 year old listening to the music generated using a LED torch in combination with a light modulated unit (LMU) and a stethoscope. Permission was granted by the parents to use this picture.

As part of GIFT-Surg public engagement aims, we applied as a team to present our research at the Bloomsbury Festival on Saturday 24 October (London, UK) [22] which theme this year was ‘On Light’. At the Bloomsbury Festival, the ‘Music-of-Light Stethoscope’ saw its first public outing (www.gift-surg.ac.uk/pictures-from-the-bloomsbury-festival/). In figure 5, an 8 year old child is learning about the PA effect with the help of our torch-and-stethoscope-based device.

The presentation of the demo was accompanied by short multimedia videos, interactive games and printed handouts which helped to explain how the same PA process is being used for advanced surgical imaging devices on the GIFT-Surg Project. The combination of the demo with the supporting materials enabled the team to explain a complex physical process to a broad audience and engage them in a real-world application for the science. It also meant the team could adapt the presentation of the work to suit the age range of the participants and the level of detail needed to keep their interest. In the stall people also had an opportunity learn more about the developed technologies by asking questions of the scientists.

**Figure 6.**
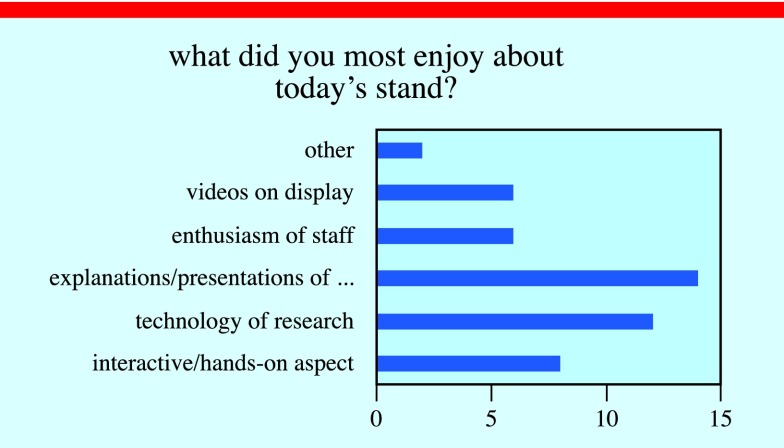
Results from the evaluation questionnaire conducted at the event on what participants most enjoyed, citing the team presentations as the favourite.

**Figure 7.**
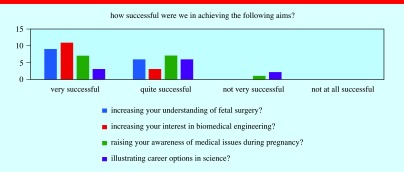
Results showing the assessment of how successful our demo stand was in promoting the sciences and the research of the project.

At the festival, we conducted in-depth evaluation interviews with groups that interacted with us for a period of time. This provided valuable and quantitative insight into how successful we were at engaging with our audience. Over 100 people visited our stall, with the two main populations being ‘under 12 s’ and ‘35–50 age’ range, which together represented family groups. Twenty families returned the questionnaires assessing what they learnt from the demos (figures 6 and 7).

The results show that families found the team presentations, technology behind the research project and the interactive nature of the demos to be the most enjoyable aspects of their visit to our stand (figure 6). In addition, all of our respondents classed the stand as being ‘Successful’ or ‘Very Successful’ at promoting Biomedical Engineering and increasing understand of the research into fetal surgery through their participation at the stand (figure 7). This evaluation exercise shows that we were successful in using our scientific demonstrations to engage the public in our work and foster a passion for the sciences in the younger generation.

Since the Bloomsbury Festival, the device has also been used to at the UCAS days [21] at UCL to promote STEM subjects to the younger generations. This demo was also presented to the funders of the GIFT-Surg Project (Wellcome Trust and EPSRC) to showcase the progress being made in its public engagement activities, which will be key to securing future funding opportunities for the University and proving its commitment, as an institute, to this area.

## 6. Discussion and future work

We developed the ‘Music-of-Light Stethoscope’ demonstration kit that uses modulated light to generate audible sound waves, which could help the general public to better understand the generation of sound using light. This forms the basis of PA imaging. Although, compared to the ultrasound generated for PA imaging, a different mechanism was responsible for the audible sound generation, our demonstration provided an intuitive solution so that people can directly hear it.

The first iteration of the system didn’t have speakers as the PA signal can be clearly heard using a stethoscope. But for demonstrating to a large audience it is not efficient to let people hear the music one by one. In addition, sharing a single stethoscope within a large group of people might present ear infection spreading risks unless disposable earplugs are used. For this reason, in the current system, we decided to implement an additional amplification stage with external speakers.

One of the problems that we encountered during the demonstration is a loud squeal or screech sound due to positive feedback between the microphone and the loudspeakers. This audio feedback is known as acoustic feedback or the Larsen effect. In order to minimize this effect, the speakers should be placed at some distance and directed away from the microphone embedded in the electronic stethoscope.

Another way of improving the demo is to design a mechanically amplified system relying, for example, on a parabolic drum having a black membrane that can be used as a PA absorbing target. This might allow avoidance of using an expensive electronic stethoscope.

At the Festival we found out that the level of PA signal played through the speakers is not always loud enough in the large and noisy open-space conference environment. To address that while improving the quality of the music, both a stronger amplifier and a higher power LED or several LEDs could be used for the generation of louder music. The ‘Music-of-Light Stethoscope’ could also be enclosed in a sound-proof but transparent enclosure to limit the feedback between the external speakers and the stethoscope chest piece.

## 7. Conclusion

In this paper we demonstrated the development of an interactive setup that uses light to transfer music through the PA effect. This demo was successfully tested at the Bloomsbury Festival 2015 *On Light* and proved to be an effective tool to teach the PA effect and increase the interest of the public in biomedical engineering. The department now has a novel device to use at future events and further promote and inspire the work of the department to the wider community.
